# HIV-1 integrase polymorphisms are associated with prior antiretroviral drug exposure

**DOI:** 10.1186/1742-4690-6-12

**Published:** 2009-02-09

**Authors:** Sebastiaan J van Hal, Belinda Herring, Zaquan Deris, Bin Wang, Nitin K Saksena, Dominic E Dwyer

**Affiliations:** 1 Centre for Infectious Diseases and Microbiology, ICPMR Westmead Hospital, University of Sydney, Westmead 2145, NSW, Australia; 2 Retroviral Genetics Division, Centre for Virus Research, Westmead Millennium Institute, Westmead 2145, NSW, Australia; 3 Universiti Sains Malaysia, Department of Medical Microbiology and Parasitology, Kota Bharu, Kelantan, Malaysia

## Abstract

In a recent summary of integrase sequences, primary integrase inhibitor mutations were rare. In a review of integrase inhibitor-naïve Australian HIV-1 sequences, primary mutations were not identified, although the accessory mutation G140S was detected. A link with previous antiretroviral therapy, intra-subtype B divergence across the integrase gene and transmission of integrase polymorphisms were also noted. Based on these findings, we would recommend ongoing surveillance of integrase mutations, and integrase region sequencing for patients prior to commencement of integrase inhibitors.

## Correspondence

We congratulate Rhee and colleagues for their extensive review of the natural variation of HIV-1 integrase in 1500 integrase sequences from B and non-B HIV-1 subtypes [1]. This study provides significant insights into integrase inhibitor (INIs) resistance mutations and polymorphisms, and forms an essential component in guiding therapeutic decisions. This has become even more imperative since the licensing of raltegravir (RAL) for the treatment of antiretroviral (ARV)-experienced HIV-1 infected patients, following the definitive BENCHMRK 1 and 2 trials [2,3].

In both BENCHMRK trials, treatment failure was associated with the selection of signature, or primary, INI mutations. These occur in one of two main pathways, either N155H or Q148H/K/R. A possible alternative and third pathway, Y143R/C, also exists. Primary INI mutations were detected by Rhee et al. in their review of the Stanford HIV Database in only 3 isolates (each with a single primary INI mutation N155H, Q148H and Q148K). We are aware of possibly two additional isolates, one from an Australian isolate bearing N155H in an INI-naïve patient (GenBank Accession No. AF042104) and a second resistant isolate published by Myers et al. [4]. Therefore, although primary mutations remain rare in INI treatment-naïve individuals, their occurrence suggests that IN sequencing should be considered in all patients prior to INI therapy.

The role of accessory mutations in INI resistance is less clear. It is known that pathway-specific accessory mutations augment INI resistance in the presence of the primary mutations. However, the phenotypic effect of most isolated single accessory mutations (i.e. T97A, V151I, G163R, I203M and S230N) remains unknown. In contrast, isolated L74A/I/M has no effect on in vitro resistance while G140S is associated with an in vitro 5–10 fold decrease in susceptibility [4] (Personal communication with Merck Sharp & Dohme). This mutation is not only responsible for partial RAL resistance but also restores viral fitness when combined with the Q148R/H mutation [5]. G140S was not documented by Rhee et al. in their review [1]. However, in a small study conducted at Westmead Hospital, Sydney, Australia, G140S was detected in 2 INI-naïve patients, as were the other accessory mutations: L74I/M (n = 8), T97A (n = 2), V151I (n = 3) and I203M (n = 4). In this study, plasma from 133 INI-naïve patients were sequenced (GenBank Accession Numbers FJ554674–FJ554806) across integrase. Most sequences belonged to HIV-1 subtype B (n = 109). The remaining 25 sequences include subtype A (n = 4); subtype C (n = 8); subtype G (n = 4), CRF02_AG (n = 3), CRF01_AE (n = 2) and CRF33_01B (n = 3). Using the same HIV-1 subtype B consensus sequence as Rhee and colleagues, similar subtype-specific consensus residues were detected. For the newly described CRF33_01B subtype (not studied by Rhee et al.) several polymorphisms were detected in all sequences i.e. V31I, T112V, T125A [6]. Although other residues were similar to other non-B subtypes (varying from the consensus B subtype), no firm conclusions can be made because the number of CRF33_01B sequences studied was small.

The influence of prior ARV therapy (not including INIs) was not addressed by Rhee et al. Interestingly, we found that previous ARV therapy was associated with greater IN divergence (the mean intra-subtype B divergence was 5.1% +/- 0.17 in 59 samples from treatment-experienced patients compared to a mean of 3.8% +/- 0.18 in 50 treatment-naïve samples; p < 0.01). This finding suggests that ARV-induced changes in other parts of the HIV-1 genome may be linked to integrase polymorphisms. This is supported by data that has detected several integrase polymorphisms (e.g. M154I, V165I and M185L) positively associated with specific RT mutations (F227L and T215Y) in samples from treated individuals [7]. We were unable to find any evidence to suggest this co-evolution at the IN and RT sites, probably as a consequence of our small sample size. It still remains unknown whether the efficacy of INIs is affected by previous ARV therapy selection pressure. However, both G140S mutations in our study occurred in samples from previously ARV-treated individuals, suggesting that previous therapy may result in reduced INI efficacy, and further supporting IN sequencing in patients contemplating INI therapy initiation.

A further question not addressed by Rhee and colleagues was the potential transmission of IN polymorphisms. In our study, three subtype B-infected patients (GenBank Accession Numbers FJ554692; FJ554718; FJ554739) were known to have acquired their infection from the same source. These samples all had IN sequencing performed on plasma, cultured isolates and peripheral blood mononuclear cells. Several IN polymorphisms (S24N, D25E, T112I, S119P, T125A, K136Q, V201I, L234I and S283G) were present in all three patients, suggesting that they were readily transmitted. Interestingly, the sequences did not vary by more than 0.2%, suggesting little sequence variation between the various HIV-1 "compartments".

In conclusion, ongoing surveillance of integrase inhibitor polymorphisms is important, including in non-subtype B viruses. We suggest that IN sequencing should be undertaken in all patients prior to INI commencement to enable further elucidation of resistance pathways, and determination of the significance of subtype-specific polymorphisms in both ARV-naïve and experienced individuals.

## Consent and ethics approval HREC 2008/3/4.10 (2750)

Patient consent was deemed unnecessary by the local Ethics committee for several reasons: Antiviral resistance testing is the standard of care for all Australian HIV infected patients. Stored samples used for previous antiviral resistance testing were available therefore no patients were approached for this study to provide new blood samples. Furthermore, it was felt that this testing could potentially benefit the patient in future when integrase inhibitors may be required in the treatment regimen.

## Competing interests

D. Dwyer is a member of the advisory boards for Merck Sharp & Dohme Australia and other local pharmaceutical companies. Dr S. van Hal has received sponsorship to attend a local HIV conference from Merck Sharp & Dohme Australia

## Authors' contributions

DED, NKS conceived the study. SJvH, BH, BW and DZ performed the sequences and subsequent analysis. SJvH wrote the paper. All authors have read and approved the final manuscript.
